# Cell-penetrating peptide-mediated cell entry of H5N1 highly pathogenic avian influenza virus

**DOI:** 10.1038/s41598-020-74604-w

**Published:** 2020-10-22

**Authors:** Naoki Kajiwara, Namiko Nomura, Masako Ukaji, Naoki Yamamoto, Michinori Kohara, Fumihiko Yasui, Yoshihiro Sakoda, Hiroshi Kida, Futoshi Shibasaki

**Affiliations:** 1 Molecular Medical Research Project, Department of Genome Medicine, Tokyo Metropolitan Institute of Medical Science, 2-1-6, Kamikitazawa, Setagaya-ku, Tokyo, 156-8506 Japan; 2 Department of Microbiology and Cell Biology, Tokyo Metropolitan Institute of Medical Science, Tokyo, Japan; 3 Laboratory of Microbiology, Department of Disease Control, Graduate School of Veterinary Medicine, Hokkaido University, Sapporo, 060-0818 Japan; 4 Present Address: Neurovirology Project, Department of Diseases and Infection, Tokyo Metropolitan Institute of Medical Science, Tokyo, Japan; 5 Present Address: Center for Medical Research Cooperation, Tokyo Metropolitan Institute of Medical Science, Tokyo, Japan

**Keywords:** Pathogens, Virology, Mechanisms of disease, Protein transport, Drug delivery

## Abstract

H5N1 highly pathogenic avian influenza virus (HPAIV) poses a huge threat to public health and the global economy. These viruses cause systemic infection in poultry and accidental human infection leads to severe pneumonia, associated with high mortality rates. The hemagglutinin (HA) of H5N1 HPAIV possesses multiple basic amino acids, as in the sequence RERRRKKR at the cleavage site; however, the role of this motif is not fully understood. Here, we showed that a 33-amino acid long peptide derived from HA of H5N1 HPAIV (HA314-46) has the potential to penetrate various cells and lung tissue through a sialic acid-independent endocytotic pathway. Mutant peptide analyses revealed that the cysteine residue at position 318 and multiple basic amino acids were essential for the cell-penetrating activity. Moreover, reassortant viruses possessing H5 HA could enter sialic acid-deficient cells, and virus internalisation was facilitated by cleavage with recombinant furin. Thus, our findings demonstrate that the HA314-46 motif exhibits cell-penetrating activity through a sialic acid-independent cell entry mechanism.

## Introduction

Influenza A viruses are responsible for the seasonal epidemics and occasional pandemics in humans and animals^1^. These viruses have a segmented, single-stranded, negative-sense RNA genome within the envelope^1^. Among many subtypes, H5N1 highly pathogenic avian influenza viruses (HPAIVs) are a serious threat to public health and the global economy^2^. Since the first human infection in Hong Kong^3,4^, H5N1 HPAIVs have caused hundreds of hospitalisations and deaths with a high fatality rate^5,6^. Furthermore, the emergence of other neuraminidase subtypes such as H5N6 and H5N8, arising from the same ancestral H5N1 virus, has threatened human and animal health^7,8^. Hence, a better understanding of the interspecies transmission mechanism is crucial to prevent and control the spread of HPAIVs with H5 subtypes.

Influenza A virus infection is initiated by the binding of hemagglutinin (HA) to cell surface receptors, sialylated glycoconjugates^9^. There are two main forms that determine the host tropism: sialic acid-α(2,3)-galactose for avian cells and sialic acid-α(2,6)-galactose for mammalian cells^10^. Following cell entry through endocytosis, the HA proteins change conformation under low pH conditions in the endosome, which causes fusion of viral-endosomal membranes^9^. For membrane fusion to occur, the precursor protein HA0 must become proteolytically cleaved into HA1 and HA2 subunits in the *trans-*Golgi network or on the plasma membrane^11^. In contrast with the HA proteins of low pathogenic viruses, the HA proteins of H5N1 HPAIVs contain multiple basic amino acids at the cleavage site^12^. This characteristic sequence is cleaved by ubiquitous cellular proteases, such as furin and proprotein convertase 6^13,14^, suggesting systemic infection in poultry^15^. Thus, the amino acid sequence of HA cleavage site is a key determinant for organ tropism and pathogenicity of influenza A viruses.

The HA cleavage site motif of H5N1 HPAIV resembles cationic cell-penetrating peptides (CPPs) such as trans-activator of transcription (TAT) from human immunodeficiency virus type 1 (HIV-1)^16^. The representative amino acid sequence of HA cleavage site motif from H5N1 HPAIV is RERRRKKR and that of TAT peptide is YGRKKRRQRRR. CPPs can efficiently transport a wide variety of biologically active conjugates including proteins, peptides, nucleic acids, small chemical compounds, and nanoparticles into cells via energy-dependent endocytosis and/or energy-independent direct penetration^17,18^. In addition to TAT peptide^19,20^, peptides with cell-penetrating activity have been found in other viruses, such as herpes simplex virus type 1^21^, dengue virus^22^, hepatitis B virus^23,24^, and human papillomavirus^25,26^. However, little is known about the functional roles of these peptides during virus infection. For example, TAT is secreted by HIV-infected cells, but the role of the secreted TAT in virus replication or pathogenesis remains elusive^27^.

In the present study, we tested the hypothesis that the cell-penetrating activity of HA cleavage site motif stimulates a sialic acid-independent cellular entry of H5N1 HPAIV.

## Results

### The C-terminal domain of HA1 protein from H5N1 HPAIV has an ability to internalise into cells

**Figure 1:**
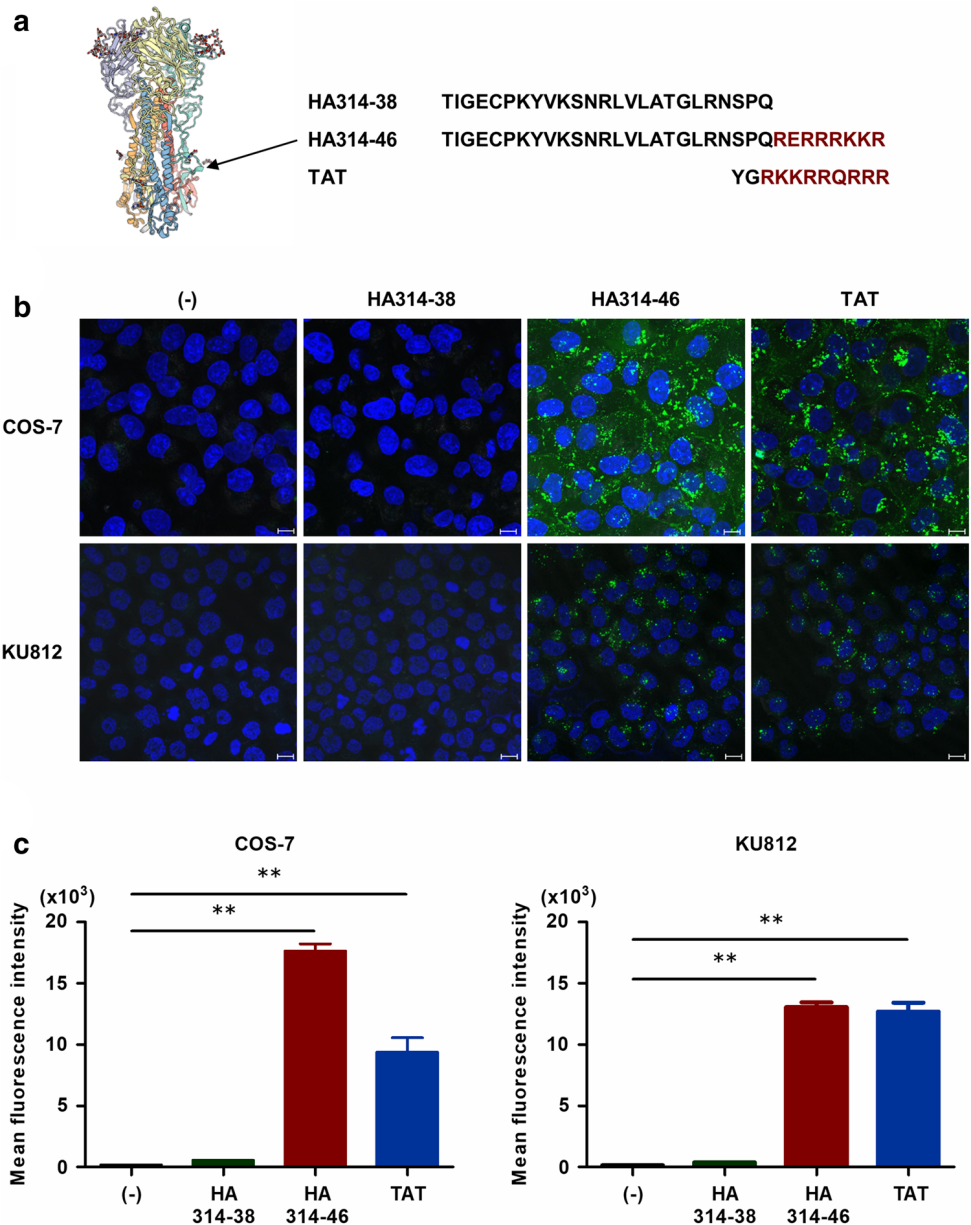
H5N1 HPAIV-derived HA314-46 peptide exhibits cell-penetrating activity. (**a**) Three-dimensional structure of HA protein and amino acid sequences of H5N1 HPAIV-derived HA314-38, HA314-46, and HIV-1-derived TAT peptides. The structure and sequence were obtained from the SWISS-MODEL Repository^50^ (https://swissmodel.expasy.org/repository/uniprot/D9I6N5) and the UniProt database (Protein ID; D9I6N5), respectively. Red-coloured residues represent multiple basic amino acids in HA314-46 and TAT peptides. (**b,c**) Cell-penetrating activity of HA314-46 peptide in COS-7 and KU812 cells. COS-7 and KU812 cells were incubated with 10 μg/mL of FITC-conjugated peptides at 37 °C for 60 min. Internalisation of the peptides was examined using confocal microscopy (**b**). Representative images from three experiments are shown. Green, peptide; Blue, nucleus. Scale bar, 10 μm. Mean fluorescence intensity (MFI) of FITC in viable cells was measured by flow cytometry (**c**). Data are presented as mean + SEM (n = 3). Asterisks indicate significant increase by one-way ANOVA with Bonferroni's multiple comparison test. **p < 0.01.

In general, the activities of CPPs are not selective^17^. We first investigated whether the C-terminal domain of H5N1 HPAIV HA1 protein has cell-penetrating activity. For these experiments, HA314-46 peptide comprising the wild-type C-terminus of HA1 protein from H5N1 HPAIV and the HA314-38 peptide lacking the multiple basic amino acids were used (Fig. 1a). TAT peptide was used as a positive control for CPP assay. These peptides were labelled with fluorescein isothiocyanate (FITC). Confocal microscopic examination of COS-7 cells incubated with HA314-46 or TAT peptide showed that the spotted fluorescence signal was localised in both the cytoplasm and nucleus (Fig. 1b and Supplementary Fig. S1). In contrast, there was no internalisation of HA314-38 peptide. To confirm the CPP activity of HA314-46 peptide, KU812 cells were also treated with these peptides. Similar to TAT, the HA314-46 peptide could also penetrate into the cells. However, HA314-38 peptide was not cell-permeable. To quantify the intensity of cell penetration, geometric mean fluorescence intensity (MFI) in viable cells was measured by flow cytometry. Cells incubated with HA314-46 or TAT peptide displayed a significant increase of MFI, while HA314-38 mutant peptide lost the ability to penetrate cells (Fig. 1c). We further evaluated the duration of peptide uptake. The HA314-46 peptide uptake was maximised at 120 min and remained until 240 min (Supplementary Fig. S2). Conversely, the cell-penetrating activity of TAT peptide peaked at 30 min. HA314-38 mutant peptide was not internalised following incubation for 240 min. Cationic CPPs are known to non-specifically bind to the outside of the cell membrane, leading to false-positive results^28^. To remove the peptides absorbed to cell surface, KU812 cells were treated with 0.1% trypsin after peptide uptake. The increase of MFI in the case of HA314-46 or TAT peptide persisted even after trypsin treatment (Supplementary Fig. S3), indicating the intracellular localisation of the peptide.

### H5N1 HPAIV-derived HA314-46 peptide exhibits a broad cell tropism

**Figure 2:**
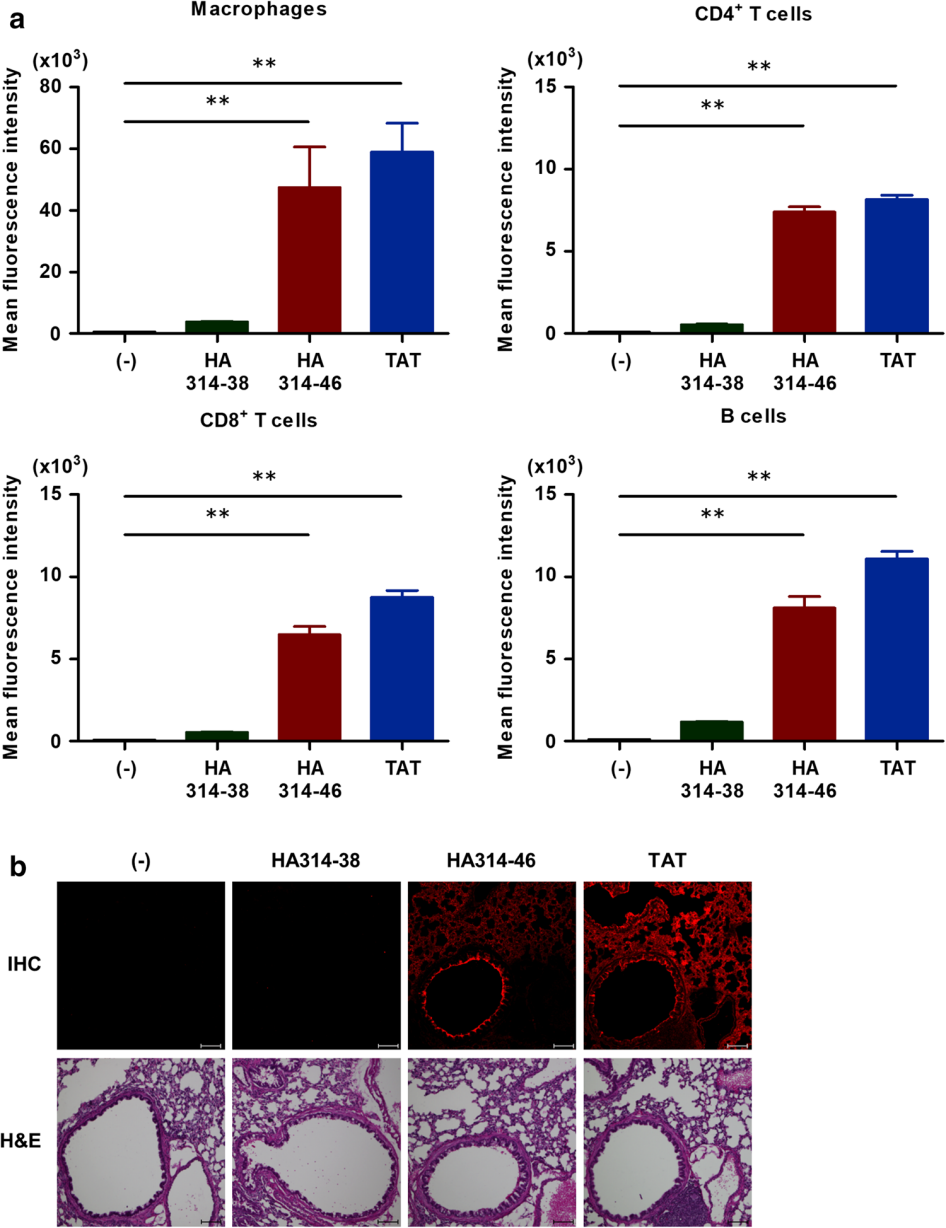
HA314-46 peptide penetrates several different types of cells and mouse lung tissue. (**a**) Cell-penetrating activity of HA314-46 peptide in mouse primary cells. Splenocytes were freshly isolated from C57BL/6 N mice and were incubated with or without 10 μg/mL of FITC-conjugated peptides for 60 min at 37 °C. MFI of FITC in each cell population was analysed by flow cytometry. Data are presented as mean + SEM (n = 4). Asterisks show significant increase by one-way ANOVA with Bonferroni's multiple comparison test. **p < 0.01. (**b**) Peptide distribution in the lung tissue of mouse intranasal inhalation model. C57BL/6 N mice were intranasally exposed with 25 μg of FITC-conjugated peptides or saline (-). Lung frozen sections were stained with anti-fluorescein/Oregon Green polyclonal antibody and Alexa Fluor 555-conjugated secondary antibody for eliminating autofluorescence in the sections. The distribution of peptides was examined by confocal microscopy. Representative images from five experiments are shown. Red, peptide. Scale bar, 100 μm. Counterstaining of the serial section was done with H&E dyes.

Next, we examined the cell specificity of H5N1 HPAIV-derived HA314-46 peptide using a wide variety of cell lines of different origins. Cells were incubated with HA314-38, HA314-46, or TAT peptides and then geometric MFI in viable cells was determined by flow cytometry. The results showed that HA314-46 peptide was non-selectively internalised into adherent (A549, HeLa, NIH/3T3, J774A.1, COS-7, and MDCK), and non-adherent (KU812, Jurkat, Raji, U937, THP-1, and HL-60) cell lines, as is the case with TAT peptide (Supplementary Table S1). In non-adherent cell lines, HA314-46 peptide was observed to be highly permeable in KU812 cells, whereas it showed low incorporation into HL-60 cells at the same dose. To investigate the incorporation of HA314-46 peptide into primary cells, mouse splenocytes were prepared and subjected to cell penetration assay. Cell surface markers were utilised for the separation of cell populations: macrophages, CD11b^+^F4/80^+^MHC class II^+^; CD4^+^ T cells, CD3^+^CD4^+^; CD8^+^ T cells, CD3^+^CD8^+^; and B cells, CD19^+^B220^+^ (Supplementary Fig. S4). These cell surface markers could be stained with antibodies even following a 0.1% trypsin treatment. Flow cytometric analysis revealed that HA314-46 peptide was able to penetrate immune cells including macrophages, CD4^+^ and CD8^+^ T cells, as well as B cells (Fig. 2a). We further tested the cell-penetrating activity of HA314-46 peptide in vivo. For this purpose, anaesthetised mice were intranasally administered with peptides to monitor the distribution in lung tissues. Tissue sections showed the wide distribution of HA314-46 and TAT peptides in lungs (Fig. 2b). In the same model, non-permeable HA314-38 peptide was not detected in the lungs as the peptide was washed out during the preparations of tissue sections.

### Cysteine residue and multiple basic amino acids are necessary for HA314-46 peptide internalisation

**Figure 3:**
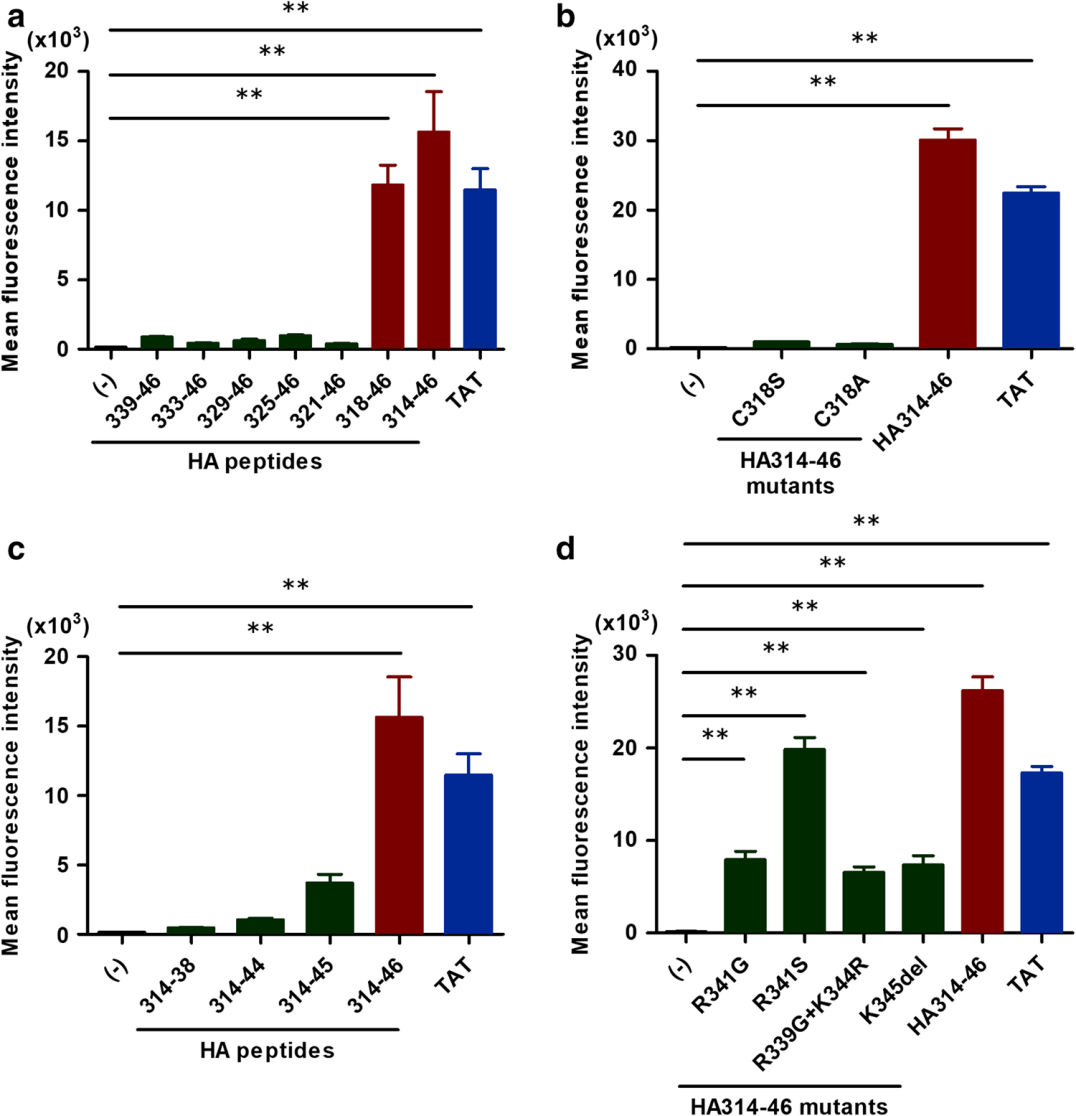
Cysteine residue and/or multiple basic amino acid mutations impair the cell-penetrating activity of HA314-46 peptide. (**a**) Screening of membrane-permeable HA peptide sequences. KU812 cells were incubated with different lengths of HA peptides or a TAT peptide. (**b**) Cell-penetrating activities of cysteine-substitution mutants of HA314-46 peptide. KU812 cells were incubated with cysteine-substitution mutants of HA314-46 (C318S or C318A), HA314-46, or TAT peptide. (**c**) Cell-penetrating activities of C-terminal basic amino acid deletion mutants of HA314-46 peptide. KU812 cells were incubated with HA314-38, HA314-44, HA314-45, HA314-46, or TAT peptides. (**d**) Cell-penetrating activities of C-terminal amino acid mutants observed in naturally occurring isolates of H5 subtype. KU812 cells were incubated with intact or mutant HA314-46 or TAT peptide. After incubation with 10 μg/mL of FITC-conjugated peptides at 37 °C for 60 min, the cells were subjected to flow cytometric analysis to measure MFI of FITC. Data are presented as mean + SEM (n = 3 or 4). Asterisks indicate significant increase by one-way ANOVA with Bonferroni's multiple comparison test. **p < 0.01.

Using several peptides, we attempted to determine the essential motif of the HA314-46 peptide. The amino acid sequence of each peptide is summarised in Table 1. In this screening, KU812 cells were exposed to HA peptides of various lengths. After washing and trypsinisation, geometric MFI was measured using flow cytometry. Two HA peptides (HA318-46 and HA314-46) permeated through the plasma membrane and resulted in an increase of MFI in viable cells (Fig. 3a). These peptides included a 29-amino acid residue region, spanning from 318 to 346 of HA protein from H5N1 HPAIV. Meanwhile, the shorter HA peptides, such as HA339-46, did not exhibit cell-penetrating activity. To identify amino acid residues essential for the activity of HA314-46 peptide, we replaced the cysteine residue at position 318 with serine (C318S) or alanine (C318A). Strikingly, C318S or C318A mutations abolished the cell-penetrating activity of HA314-46 peptide (Fig. 3b). In UniProt protein knowledgebase (Q5EP31), cysteine residues are known to form disulphide bonds at position 294 and 318. To determine whether disulphide bond formation affects the cell-penetrating activity, HA peptides were incubated in the presence or absence of dimethyl sulfoxide (DMSO)^29^. In contrast to the C318S- and C318A-mutant peptides, HA314-38 and HA314-46 peptides produced homodimers by DMSO oxidation (Supplementary Fig. S5a and S5b). Mass spectrometry analyses showed that the peak area ratio of homodimer/monomer in HA314-46 peptide was increased by DMSO oxidation (DMSO (-) 0.11 vs DMSO ( +) 0.91). In HA314-46 (C318S) mutant peptide, no difference was observed in peak area ratio of homodimer/monomer between DMSO (-) and DMSO ( +). DMSO-treated HA314-46 peptide exhibited a twofold increase in MFI compared to the non-treated peptide (Supplementary Fig. S5c), indicating the uptake of homodimers containing two FITC molecules.

**Table 1:** List of peptides for CPP assay.

Peptides	Amino acid sequences
HA314-46	TIGE**C**PKYVKSNRLVLATGLRNSPQ**RERRRKKR**
HA339-46	**RERRRKKR**
HA333-46	LRNSPQ**RERRRKKR**
HA329-46	LATGLRNSPQ**RERRRKKR**
HA325-46	NRLVLATGLRNSPQ**RERRRKKR**
HA321-46	YVKSNRLVLATGLRNSPQ**RERRRKKR**
HA318-46	**C**PKYVKSNRLVLATGLRNSPQ**RERRRKKR**
HA314-46 (C318S)	TIGESPKYVKSNRLVLATGLRNSPQ**RERRRKKR**
HA314-46 (C318A)	TIGEAPKYVKSNRLVLATGLRNSPQ**RERRRKKR**
HA314-38	TIGE**C**PKYVKSNRLVLATGLRNSPQ
HA314-44	TIGE**C**PKYVKSNRLVLATGLRNSPQ**RERRRK**
HA314-45	TIGE**C**PKYVKSNRLVLATGLRNSPQ**RERRRKK**
HA314-46 (R341G)	TIGE**C**PKYVKSNRLVLATGLRNSPQ**REGRRKKR**
HA314-46 (R341S)	TIGE**C**PKYVKSNRLVLATGLRNSPQ**RESRRKKR**
HA314-46 (R339G+K344R)	TIGE**C**PKYVKSNRLVLATGLRNSPQ**GERRRRKR**
HA314-46 (K345del)	TIGE**C**PKYVKSNRLVLATGLRNSPQ**RERRRKR**
TAT	YG**RKKRRQRRR**
R9	**RRRRRRRRR**

Cysteine residue and multiple basic amino acids are shown in bold font. The underlined text indicates the mutation sites observed in naturally occurring isolates of H5 subtype.

Next, we examined the functional importance of multiple basic amino acids in greater detail. For this experiment, we deleted one or two basic amino acids from the C-terminus of HA314-46 peptide and found that the cell-penetrating activity of HA314-46 was markedly decreased (Fig. 3c). Specifically, it was reduced to one-third in HA314-45 peptide, while HA314-44 peptide was unable to permeate cells. In addition, we investigated whether HA314-46 activity was affected by the mutations of multiple basic amino acids found in naturally occurring isolates of H5 subtype. All HA314-46 mutant peptides retained the ability to enter cells, yet the cell-penetrating activities were attenuated by the diverse mutations (Fig. 3d).

### HA314-46 peptide utilises endocytosis to penetrate cells

**Figure 4:**
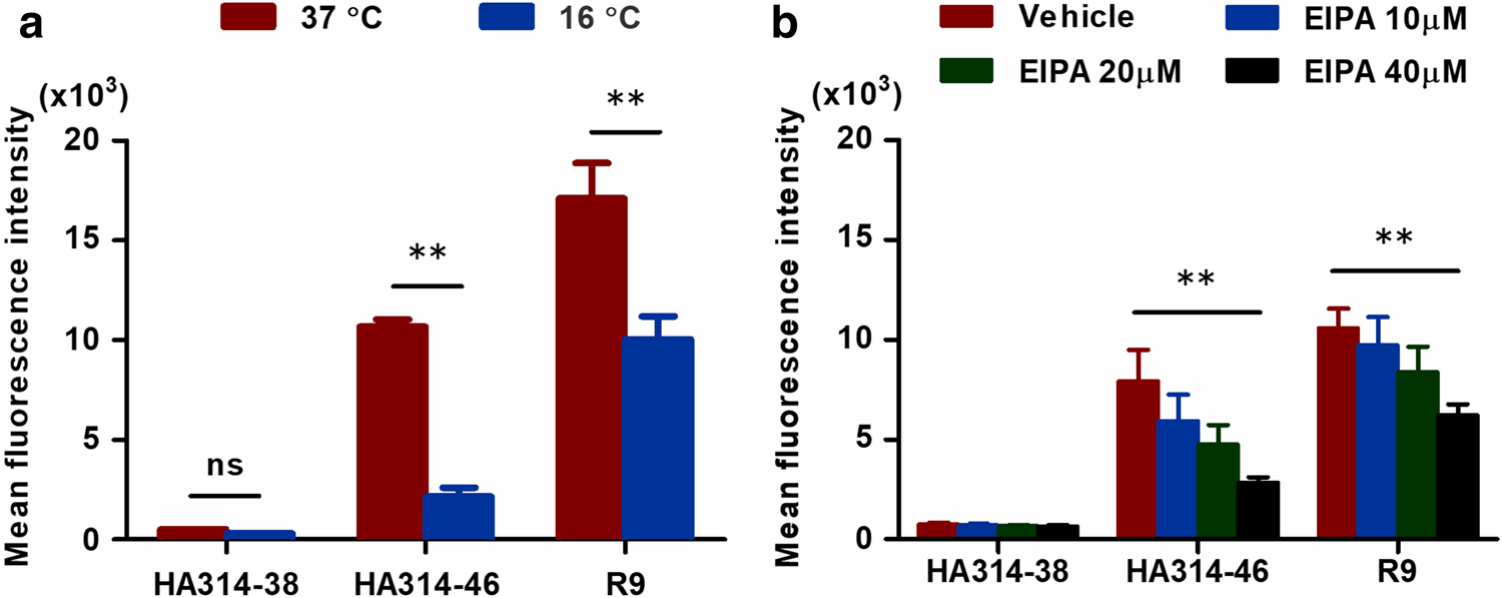
HA314-46 peptide is internalised through an energy-dependent endocytotic pathway. (**a**) Influence of incubation temperature on HA314-46 peptide uptake. KU812 cells were incubated with 10 μg/mL of FITC-conjugated HA314-38, HA314-46, or R9 peptide for 60 min at 37 or 16 °C. (**b**) Effect of EIPA on HA314-46 peptide uptake. KU812 cells were pre-treated with vehicle (DMSO) or EIPA (10, 20, 40 μM) for 30 min at 37 °C. Then, the cells were incubated with 10 μg/mL of FITC-conjugated HA314-38, HA314-46, or R9 peptide for 60 min at 37 °C. MFI of FITC in viable cells was analysed by flow cytometry. Data are presented as mean + SEM (n = 3 or 7). Asterisks indicate significant decrease by two-way ANOVA with Bonferroni's multiple comparison test. **p < 0.01; ns, not significant.

Next, we examined the cellular uptake mechanism of HA314-46 peptide. To determine whether this peptide is internalised by endocytosis or direct penetration, KU812 cells were incubated with peptides at 37 and 16 °C. The internalisation of HA314-46 peptide was markedly reduced at 16 °C, which was consistent with the case of nona-L-arginine (R9), the prototype of cationic CPP (Fig. 4a). To reveal the major endocytic pathway of HA314-46 uptake, we tested the effect of 5-(*N*-Ethyl-*N*-isopropyl) amiloride (EIPA), a macropinocytosis inhibitor. When the cells were treated with EIPA, the cell-penetrating activity of HA314-46 was inhibited in a dose-dependent manner (Fig. 4b). Similarly, the cell-penetrating activity of R9, which is partially mediated by macropinocytotic pathway^30^, was also inhibited by EIPA.

### Sialic acids are not necessary for HA314-46 peptide internalisation

**Figure 5:**
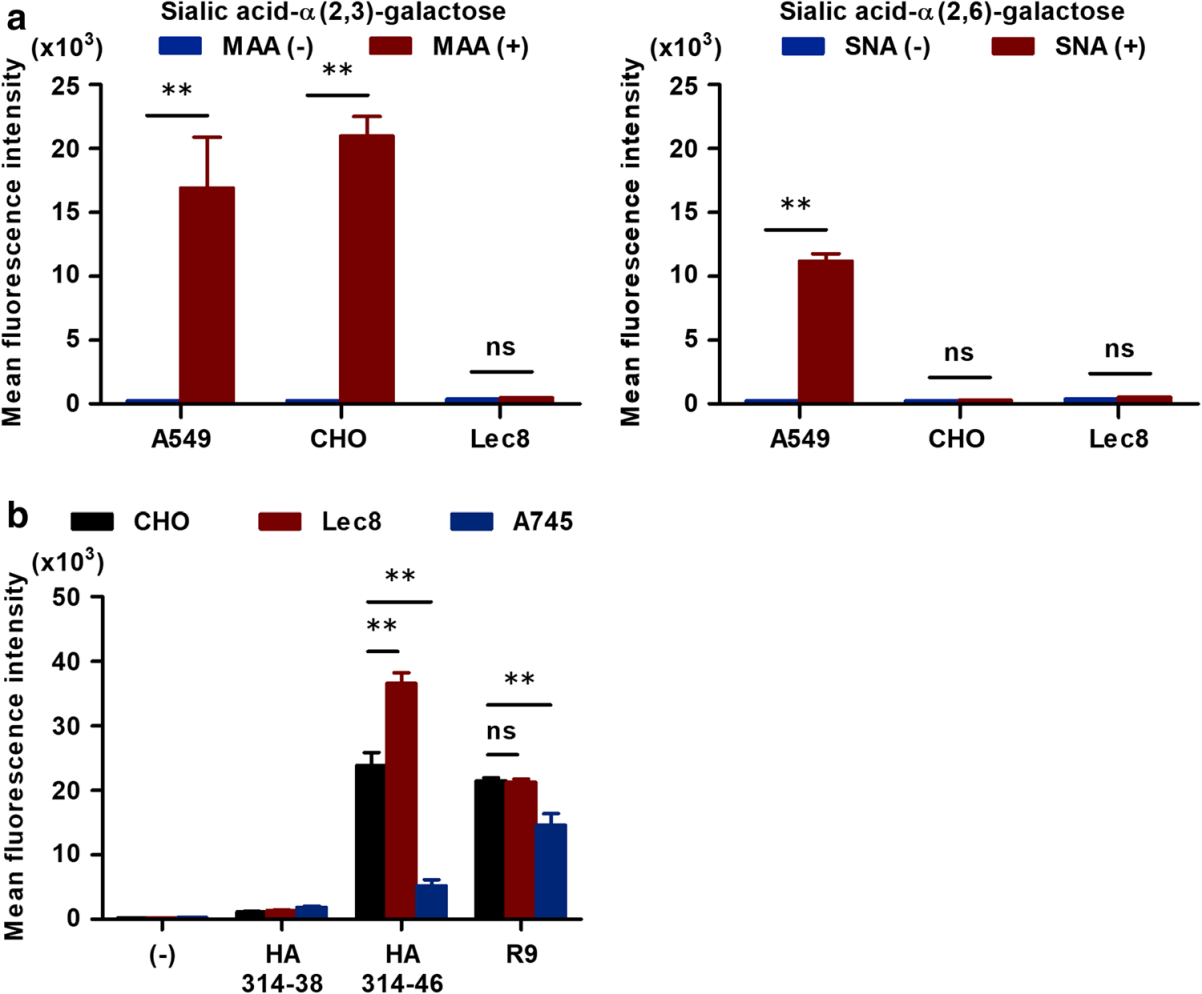
HA314-46 peptide internalisation is unconstrained by sialic acid residues. (**a**) Cell surface levels of α(2,3)- and α(2,6)-linked sialic acid-galactose on A549, CHO-K1 and Lec8 cells. The cells were stained with FITC-conjugated MAA or SNA lectin for 15 min at 24–28 °C. (**b**) Cell-penetrating activity of HA314-46 peptide in CHO-K1, Lec8 and pgsA-745 cells. These cells were incubated with 10 μg/mL of FITC-conjugated HA314-38, HA314-46, or R9 peptide for 60 min at 37 °C. MFI of FITC in viable cells was determined by flow cytometry. Data are presented as mean + SEM (n = 3). Asterisks indicate significant difference by two-way ANOVA with Bonferroni's multiple comparison test. **p < 0.01; ns, not significant.

We further investigated the uptake mechanism of HA314-46 peptide using sialidase and sialic acid-deficient cells. To determine the levels of cell surface sialic acids, which bind to influenza HA proteins, KU812 cells were stained by *Maackia amurensis* (MAA) lectin for sialic acid-α(2,3)-galactose or *Sambucus nigra* (SNA) lectin for sialic acid-α(2,6)-galactose. MFI was significantly increased in the cells stained with MAA lectin, but not SNA lectin (Supplementary Fig. S6a). Consequently, KU812 cells predominantly express sialic acid-α(2,3)-galactose on the cell surface. Next, KU812 cells were pre-treated with bacterial sialidase to test whether sialic acid residues are associated with HA314-46 incorporation. As expected, sialidase treatment led to a marked reduction in MAA-binding (Supplementary Fig. S6b), showing that α2,3-linked sialic acids were removed from the cell surface. However, the cell-penetrating activity of HA314-46 was not blocked by desialylation with sialidase (Supplementary Fig. S6c). To confirm these findings, CHO-K1 and Lec8 mutant cell lines were used. We also utilised A549 cells as a positive control for lectin staining^31^. In CHO-K1 cells, high level of sialic acid-α(2,3)-galactose was expressed, while sialic acid-α(2,6)-galactose was not detected on the cell surface (Fig. 5a). The expressions of these sialic acid residues were absent in Lec8 cells, which is defective in the transport of UDP-galactose^32^. When these cells were incubated with HA314-46 or R9 peptides, peptide uptake was not decreased in Lec8 cells (Fig. 5b). Following these experiments, we assessed the effect of trypsin pre-treatment on HA314-46 incorporation. Pre-treatment with TPCK-trypsin inhibited the uptake of HA314-46 peptide in a dose-dependent manner (Supplementary Fig. S7). Proteoglycans play a critical role in the cellular uptake of oligo-arginine peptide^33^; and to evaluate that role, we used pgsA-745 cell line which is deficient in xylosyltransferase^34^ and does not produce proteoglycans. In comparison to CHO-K1 cells, HA314-46 and R9 uptakes were greatly reduced in pgsA-745 cells (Fig. 5b).

### HA314-46 activity promotes entry of H5-subtype reassortant virus into cells

**Figure 6:**
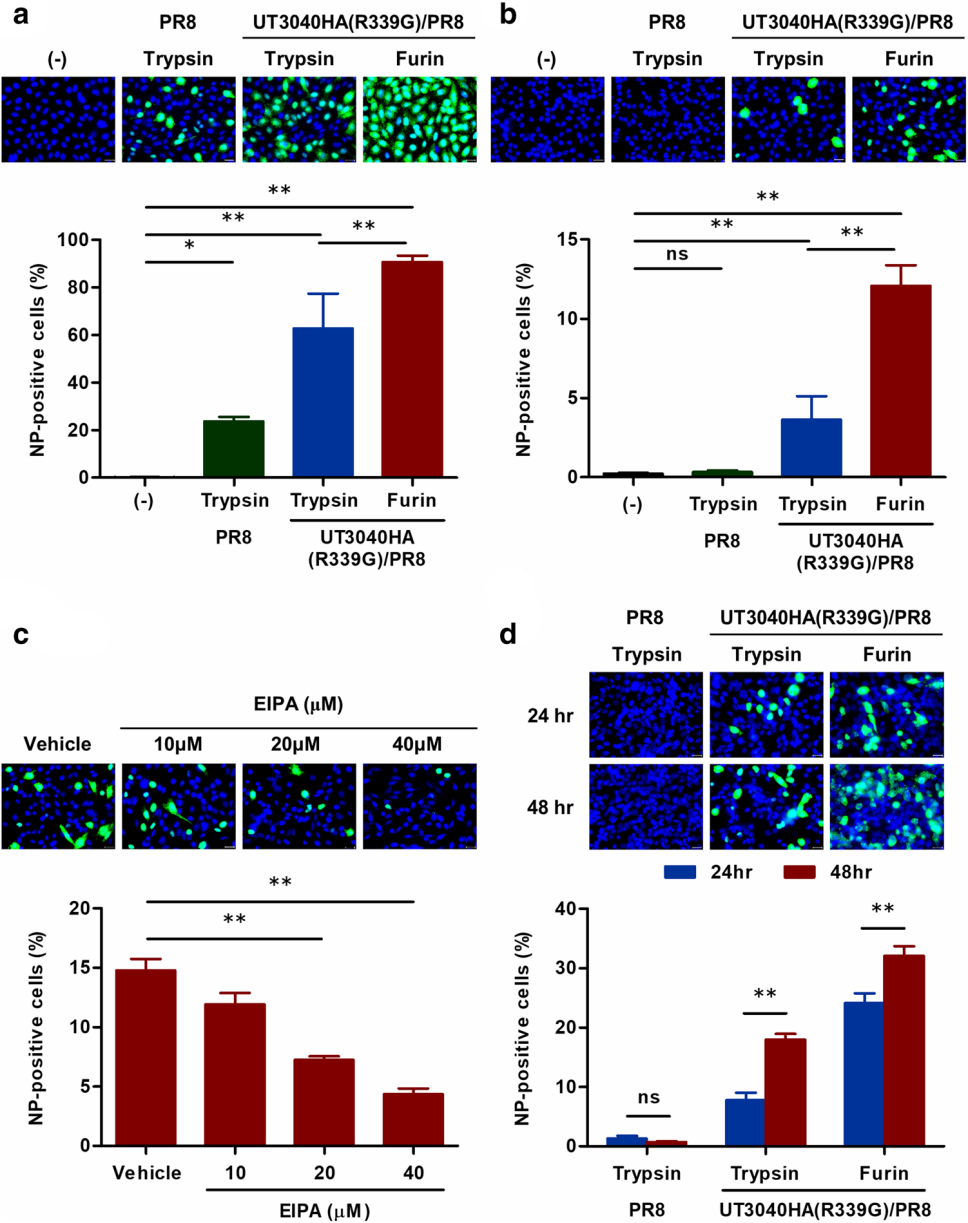
Cell-penetrating activity of HA314-46 drives sialic acid-independent virus internalisation. (**a, b**) Entry of reassortant viruses into CHO-K1 and Lec8 cells. CHO-K1 (**a**) and Lec8 (**b**) cells were incubated with trypsin- or furin-treated reassortant viruses at 37 °C for 1 h at MOI of 10. After washing and incubation for 8 h in the culture medium, the cells were fixed in 4% paraformaldehyde. (**c**) Effect of EIPA on sialic acid-independent entry of furin-treated UT3040HA (R339G)/PR8 viruses. Lec8 cells were pre-treated with vehicle (DMSO) or EIPA (10, 20, 40 μM) for 30 min at 37 °C and then were incubated with furin-treated UT3040HA (R339G)/PR8 viruses as described in (**b**). After washing, the cells were further incubated for 8 h in the culture medium containing vehicle or EIPA. (**d**) Propagation of UT3040HA (R339G)/PR8 virus in Lec8 cells. Lec8 cells were treated with reassortant viruses as described in (**b)**. After washing, the cells were incubated in the culture medium at 37 °C for 24 and 48 h. The fixed cells were permeabilised and stained with anti-influenza A nucleoprotein monoclonal antibody (**a-d**). Localisation of the viruses was evaluated by fluorescent microscopy. Representative images from four or six experiments are shown. Green; viral nucleoprotein, Blue; nucleus. Scale bar, 20 μm. The graphs in the bottom panels indicate the proportion of viral nucleoprotein-positive cells by flow cytometric analysis. Data are presented as mean + SEM (n = 4 or 6). Asterisks indicate significant difference by one-way ANOVA with Bonferroni's multiple comparison test. **p < 0.01; *p < 0.05; ns, not significant.

Next, we examined the role of HA314-46 activity in H5-subtype influenza virus infection. Several reassortant viruses were generated by reverse genetics (Table 2). PR8, comprising all A/Puerto Rico/8/1934 (H1N1) genes, served as a parental control virus. UT3040HA (R339G)/PR8 carried an R339G-mutated HA gene of the A/Vietnam/UT3040/2004 (H5N1) virus and the remaining genes of A/Puerto Rico/8/1934 (H1N1). These viruses were expanded in MDCK cells in the presence of exogenous trypsin or furin. Furin cleaves many protein precursors at the C-terminus of a consensus sequence (R-X-R/K-R)^11^. Hence, the arginine residue at position 339 of UT3040HA was replaced with glycine to avoid being cleaved at the centre of multiple basic amino acids by furin. In UT3040HA (R339G)/PR8 viruses, trypsin would cut multiple basic amino acids at random, whereas furin was predicted to cleave precisely at position 346. Using CHO-K1 and Lec8 cells, we investigated the ability of reassortant virus to invade these cells. As expected, fluorescent microscopic and flow cytometric analyses showed that CHO-K1 cells were susceptible to all reassortant viruses (Fig. 6a). In comparison with trypsin-treated UT3040HA (R339G)/PR8 virus, viral nucleoprotein-positive cells were elevated in furin-treated one. A similar result was obtained when Lec8 cells were incubated with the H5-subtype reassortant viruses (Fig. 6b). Furin processing significantly enhanced the internalisation of UT3040HA (R339G)/PR8 virus compared to those with trypsin digestion. Although sialic acid-deficient Lec8 cells were highly resistant to invasion of PR8, UT3040HA (R339G)/PR8 viruses were able to enter these cells.

**Table 2:** Origin and partial amino acid sequences of HA proteins of reassortant viruses.

Reassortant viruses	HA origins	Proteases	C-terminal amino acid sequences of HA1
PR8	A/Puerto Rico/8/34 (H1N1)	Trypsin	RSAKLRMVTGLRNIPSIQSR/
UT3040HA (R339G)/PR8	A/Vietnam/UT3040/04 (H5N1)	Trypsin	KSNRLVLATGLRNSPQ**GER**/**RR**/**K**/**K**/**R**/
UT3040HA (R339G)/PR8	A/Vietnam/UT3040/04 (H5N1)	Furin	KSNRLVLATGLRNSPQ**GERRRKKR**/

Multiple basic amino acids critical for CPP activity are shown in bold font. The underlined letters and slashes indicate the mutation site at position 339 and the predicted sites of proteolytic cleavage, respectively.

We further investigated the role of proteoglycans in the UT3040HA (R339G)/PR8 virus entry using the pgsA-745 mutant cell line. The percentage of nucleoprotein-positive cells was not impaired in pgsA-745 cells (Supplementary Fig. S8). Next, we examined the effect of EIPA on the entry of furin-treated UT3040HA (R339G)/PR8 virus into Lec8 cells. The analyses using fluorescent microscopy and flow cytometry indicated that the nucleoprotein-positive cells were significantly reduced in the presence of more than 20 μM EIPA (Fig. 6c). Total cell number and cell viability were not affected by EIPA treatment (Supplementary Fig. S9a).

To determine if reassortant viruses can replicate in Lec8 cells, the virus-inoculated cells were cultured in serum-containing medium for 24 and 48 h. In this experiment, Lec8 cells became confluent at 24 h and total cell number was comparable between 24 and 48 h cultures (Supplementary Fig. S9b). PR8 could not proliferate in this condition as HA protein was not cleaved. In contrast, exposure of UT3040HA (R339G)/PR8 to Lec8 cells considerably expanded the proportion of nucleoprotein-positive cells at 48 h post-infection (Fig. 6d), indicating that UT3040HA (R339G)/PR8 viruses are able to replicate in Lec8 cells and infectious progeny viruses are released these cells.

## Discussion

Here, we demonstrated that HA314-46, 33-amino acids in the C-terminus of H5N1 HPAIV HA1 protein, functioned as a CPP in vitro and in vivo. Moreover, both the cysteine residue at position 318 and multiple basic amino acids were of determined to be of critical importance for cell-penetrating activity. We also demonstrated that HA314-46 activity might contribute to H5N1 HPAIV entry through sialic acid-independent endocytotic pathway. To our knowledge, this is the first report to demonstrate that CPP is involved in the cellular entry of influenza A virus.

CPPs are short protein fragments that can pass through cell membranes and tissue barriers^17^. Since their first discovery in 1988, numerous CPPs have been reported^35^. However, there are several pitfalls in this field. One of the most critical issues is the insufficient experimental methods to evaluate the localisation and amount of uptake^36,37^. For example, flow cytometry is frequently used to quantitatively measure the uptake of peptides, however, it is difficult to discriminate between internalised and cell surface bound peptides using only this method. Here, using two different techniques, namely confocal microscopy and flow cytometry, we showed here that the C-terminal domain of H5N1 HPAIV HA1 protein functioned as a CPP. In this study, the HA314-46 internalisation was further confirmed using 0.1% Trypsin–EDTA treatment for 10 min at 37 °C after peptide incubation to circumvent the non-specific binding of the peptides on the cell surface. Although our findings were inconsistent with those of a recent brief report that described the shorter sequence (SPQRESRRKKR) of H5 HA cleavage site acts as a CPP^38^, the controversial results may have been due to their evaluation of cellular uptake using flow cytometry without efficient treatment with a protease, such as trypsin. The discrepancy should be carefully evaluated in further studies. Additionally, in a screening assay for HA peptide with cell-penetrating activity, we demonstrated that the cysteine residue played a pivotal role in the cell-penetrating activity of HA314-46. Although our findings indicate that disulphide-linked HA314-46 homodimers also possess the cell-penetrating activity, the functional role of the cysteine residue at position 318 remains unclear.

The cellular uptake mechanisms of CPPs are the subject of intense investigation^37^. First, we conducted a temperature dependent assay to assess the involvement of endocytosis. Similar to R9, the cell-penetrating activity of HA314-46 was attenuated at 16 °C which blocks energy-dependent processes without affecting membrane fluidity^36,39^. Moreover, HA314-46 internalisation was inhibited by the treatment of EIPA. Together, these results suggest that the cell-penetrating activity of HA314-46 is mediated by an energy-dependent endocytic pathway, such as macropinocytosis. Cationic CPPs have been thought to electrically interact with anionic plasma membrane components such as proteoglycans^33^. In addition, two reports that show interaction of oligo-arginine with CXC chemokine receptor type 4^40^ and binding of CendR peptide to neurophilin-1^41^ must be noted. In our study, HA314-46 internalisation is not abolished in sialic acid-deficient cells, however, it is markedly reduced by trypsin pre-treatment of cell surface. These results suggest the existence of proteinaceous receptors on the host cells for HA314-46. Although HA314-46 is able to penetrate into various types of cells, there was marked difference in uptake efficiency between KU812 and HL-60 cells. This result may be useful in identifying the specific receptor. In the present study, proteoglycans were associated with HA314-46 peptide uptake, however, it remains uncertain whether proteoglycans act as a host cell receptor in the sialic acid-independent cellular entry of H5N1 HPAIV. The reason might be the abundance of sialic acid-α(2,3)-galactose on pgsA-745 cells. The interactions of proteoglycans with HA314-46 during H5N1 HPAIV infection may be covered by the predominant pathway through sialic acid on the cell surface. Therefore, this warrants further investigation to fully elucidate the cellular entry mechanism of HA314-46.

The mechanism of viral transmission has been garnering attention in influenza virus research^42^. In H5N1 HPAIVs, many studies have focused on the affinity and specificity of HA for sialic acid receptor^42–44^. However, the tropism of H5N1 avian influenza viruses does not necessarily correlate with the distribution pattern of sialic acid^45,46^. Moreover, H5N1 viruses are shown to have an ability to infect sialidase-treated human bronchial epithelial cells^47^. H5N1 HPAIV viruses can also infect epithelial cells of the human upper respiratory tract, such as nasopharynx, adenoid and tonsil, despite the lack of sialic acid-α(2,3)-galactose^48^. These reports suggest that other viral factors may affect the transmission of H5N1 avian influenza viruses. Using reassortant virus with H5 HA and recombinant furin, we found that the cell-penetrating activity of HA314-46 might contribute to sialic acid-independent cellular entry of H5N1 HPAIV. The reason why this alternative entry route has not been noted before, might be accounted for by the partial cleavage of HA314-46 sequence by proteases at the multiple sites or the total degradation; that is, to exert the cell-penetrating activity of HA314-46 during H5N1 HPAIV infection, multiple basic amino acids, which are crucial domains for cell-penetrating activity, need to be cleaved accurately at the C-terminus of HA1 protein (RRRKKR/GLF) by proteases such as furin. Another possible reason may be the location of the cleavage site within the HA protein. Although the cleavage site protrudes from the surface, it is located in a prominent loop distant from the receptor binding site in the globular head region^11^. Similar to HA314-46 peptide, the alternative virus entry pathway occurred by endocytosis, and virus replication was observed in sialic acid-deficient cells. Therefore, our findings propose a sialic acid-independent mechanism in which H5N1 HPAIV invades host cells by using CPP activity. Additional studies expanding our findings to animal models are needed to understand the significance of HA314-46-mediated cellular entry in the infectivity and pathogenicity of H5N1 HPAIVs.

In conclusion, we discovered that H5N1 HPAIV has the potential to infect host cells by using CPP activity of the C-terminal domain of the HA1 protein. These findings provide novel insights into the role of HA cleavage site motif during virus infection and may help us to better understand the mechanism of H5N1 HPAIV infection.

## Methods

### Peptides

All peptides were synthesised and purified to greater than 95% for in vitro and in vivo applications (Toray Research Center, Inc., Kamakura, Japan). The qualities were confirmed using reverse-phase high-performance liquid chromatography and mass spectrometry. For detection of peptide internalisation, FITC was conjugated at the N-terminus of a peptide through a 6-aminohexanoic acid linker. FITC-conjugated peptides were dissolved in sterile deionised water at 1 mg/mL, aliquoted and stored at -80 °C until use.

### Cells

Cell lines were purchased from JCRB Cell Bank (Osaka, Japan), RIKEN BRC (Tsukuba, Japan) or ATCC (Manassas, VA, USA). COS-7, A549, MDCK, and 293 T cells were maintained in Dulbecco’s modified Eagle medium (DMEM) (Nacalai Tesque, Inc., Kyoto, Japan) supplemented with 10% foetal bovine serum (FBS), 100 unit/mL penicillin, 100 μg/mL streptomycin and 1 mM sodium pyruvate (Gibco, Grand Island, NY, USA). KU812 cells were cultured in RPMI-1640 (Gibco) supplemented with 10% FBS, 100 unit/mL penicillin, and 100 μg/mL streptomycin. CHO-K1, Lec8, and pgsA-745 cells were maintained in minimum essential medium eagle, alpha modification (α-MEM) (Nacalai Tesque, Inc.) supplemented with 10% FBS, 100 unit/mL penicillin and 100 μg/mL streptomycin. These cells were cultured at 37 °C in 5% CO_2_. In the experiments using mouse primary cells, splenocytes were freshly isolated from surgically excised spleens of naïve C57BL/6 N mice (CLEA Japan, Inc., Tokyo, Japan). All mice experiments were approved by the animal experiment committee of the Tokyo Metropolitan Institute of Medical Science (Permission number: 13075) and were conducted in accordance with the ethical guidelines for animal experiments of the Tokyo Metropolitan Institute of Medical Science.

### Confocal microscopy imaging

COS-7 cells (6 × 10^4^ cells) were cultured overnight on glass slides (Matsunami Glass Ind. Ltd., Osaka, Japan) using flexiPERM slide (SARSTEDT AG & Co. KG, Numbrecht, Germany). After washing with Dulbecco's phosphate-buffered saline (D-PBS), cells were incubated with 10 μg/mL FITC-labelled peptides or serum-free Opti-MEM (Gibco) for 60 min at 37 °C. Nuclei were stained with Hoechst 34580 (Sigma-Aldrich, St Louis, MO, USA). After washing, the localisations of fluorescent peptides were observed in living cells without fixing. KU812 cells (2.5 × 10^5^ cells) were incubated with 10 μg/mL FITC-labelled peptides or Opti-MEM in 96-well plates for 60 min. To remove peptides non-specifically bound on cell surface, cells were treated with 0.1% trypsin–EDTA for 10 min at 37 °C. After washing, cytospin slides were prepared and mounted with ProLong Gold Antifade Mountant with DAPI (Molecular Probes, Inc., Eugene, OR, USA). Slides were examined by a LSM710 confocal laser scanning microscope (ZEISS, Oberkochen, Germany). Images were processed using the ZEN 2.3 SP1 software version 14 (https://www.zeiss.co.jp/microscopy/products/microscope-software/zen.html).

### Flow cytometric analysis

COS-7 cells (2 × 10^4^ cells) were cultured in 24-well plates for one day. CHO-K1, Lec8 and pgsA-745 cells (3 × 10^4^ cells) were grown in 24-well plates for two days. After washing with D-PBS, these adherent cells were incubated with 10 μg/mL FITC-labelled peptides or Opti-MEM for 60 min at 37 °C and then harvested by 0.25% trypsin–EDTA treatment. KU812 cells and splenocytes (2.5 × 10^5^ cells) were incubated with 10 μg/mL FITC-labelled peptides or Opti-MEM in 96-well plates for 60 min and then treated with 0.1% trypsin–EDTA for 10 min at 37 °C. After Fc-receptor blocking by incubation with anti-mouse CD16/CD32 (BD Biosciences, San Jose, CA, USA), splenocytes were stained with antibodies against CD8α, CD19, B220, F4/80, I-A/I-E (BioLegend, San Diego, CA), CD3e, CD4, and CD11b (BD Biosciences) for 30 min on ice. For experiments with macropinocytosis inhibitor, KU812 cells were treated with 10, 20, and 40 μM EIPA for 30 min at 37 °C and then incubated with 10 μg/mL FITC-labelled peptides in the presence of EIPA. Data were obtained using BD FACSCantoII flow cytometer (BD Biosciences) and geometric MFI of FITC in 7-aminoactinomycin D (Sigma-Aldrich)-negative viable cells was analysed using FlowJo software version 10 (https://www.flowjo.com/solutions/flowjo) (Tree Star, Inc., Ashland, OR, USA).

### Immunohistochemical analysis of murine intranasal inhalation model

Isoflurane-anaesthetised C57BL/6 N mice were challenged with intranasal administration of 25 μg of FITC-conjugated peptides or saline. Lung tissues were isolated at 15 min after inhalation and fixed in 10% formalin neutral buffer solution (Wako Pure Chemical Industries, Ltd., Osaka, Japan). The tissues were embedded in O.C.T. Compound (Sakura Finetek Japan Co. Ltd., Tokyo, Japan) and sectioned continuously at 10-μm thickness. After washing, sections were permeabilised with 0.3% PBS-T, blocked with normal rabbit serum, and stained with Fluorescein/Oregon Green polyclonal antibody and Alexa Fluor 555-conjugated donkey anti-rabbit IgG (H + L) highly cross-adsorbed secondary antibody (Molecular Probes) for 60 min at 24–28 °C. Serial sections were counterstained with Haematoxylin and Eosin (H&E) dyes (Wako Pure Chemical Industries, Ltd.). Fluorescent images and bright-field pictures were obtained using an LSM710 confocal laser scanning microscope and a BZ-9000 microscope (KEYENCE Corp., Osaka, Japan), respectively.

### Sialic acid-galactose levels on cell surface

A549, CHO-K1, and Lec8 cells were stained with FITC-conjugated MAA or SNA lectin (EY Laboratories, Inc., San Mateo, CA, USA) at 24–28 °C for 15 min. Data were acquired using BD FACSCantoII flow cytometer and analysed using FlowJo software version 10.

### Generation of reassortant viruses by reverse genetics

HA gene of A/Vietnam/UT3040/2004 (H5N1) virus was cloned to generate reassortant viruses by reverse genetics. Viral RNA was extracted from culture supernatant containing infectious virus using ISOGEN II (NipponGene Co., Ltd. Tokyo, Japan). The cDNA was prepared by reverse transcription using SuperScript VILO cDNA Synthesis kit (Life Technologies, Carlsbad, CA, USA). Then, full-length HA gene of A/Vietnam/UT3040/2004 was amplified with gene-specific universal primer sets as described by Hoffmann et al*.*^49^. The amplified HA gene was directly cloned into BsmBI-digested pHW2000 expression vector using the In-Fusion HD Cloning kit (Takara Bio USA, Inc. Mountain View, CA, USA) according to the manufacturer’s instructions. The pHW2000 vector for the expression of A/Vietnam/UT3040/2004 HA mutant (UT3040HA (R339G)) was generated by PCR-based mutagenesis. All constructs were sequenced and analysed with ABI PRISM 3100 Genetic Analyser (Applied Biosystems, Foster City, CA, USA).

For generating reassortant viruses and parental A/Puerto Rico/8/1934 (H1N1) virus, MDCK and 293 T cells were co-transfected with eight pHW2000 vectors (1 μg of each vector) using 16 μL of TransIT-293 Reagent (Takara Bio USA, Inc.). Twenty-four hours after transfection, Opti-MEM containing 1 μg/mL trypsin acetylated from bovine pancreas (Sigma-Aldrich) or recombinant human furin (Peprotech, Rocky Hill, NJ, USA) was added, and the cells were incubated for 48 h at 37 °C. The collected viruses were further propagated in MDCK cells grown in DMEM (Nissui Pharmaceutical Co., Ltd., Tokyo, Japan) supplemented with 1 μg/mL trypsin acetylated from bovine pancreas or recombinant human furin and 1% bovine serum albumin (Sigma-Aldrich) at 37 °C for 48 h. The culture supernatants were titrated by plaque assay using MDCK cells and were stored in aliquots at -80 °C until use. HA genes of the collected viruses were sequenced to verify the absence of undesired mutations. All procedures using reassortant viruses were performed in biosafety level 3 facilities by personnel wearing powered air-purifying respirators.

### Virus infection assay

CHO-K1 and Lec8 cells (2 × 10^4^ cells) were cultured for three days in 12-well plates. These cells were incubated with reassortant viruses at an MOI 10 for 60 min at 37 °C. After washing, the cells were cultured in α-MEM containing 10% FBS, 100 unit/mL penicillin, and 100 μg/mL streptomycin for 8, 24, or 48 h. For the macropinocytosis inhibition study, Lec8 cells were pre-incubated with 10, 20, and 40 μM EIPA for 30 min at 37 °C and infected with furin-treated UT3040HA (R339G)/PR8 reassortant virus as mentioned above. Cells were incubated for an additional 8 h in the presence or absence of EIPA. The infected cells were fixed in 4% paraformaldehyde, permeabilised with 0.5% Triton-X for immunofluorescence or BD Perm/Wash buffer (BD Biosciences) for flow cytometry, and stained with anti-influenza A nucleoprotein antibody (Clone HB65) and Alexa Fluor 488-conjugated goat anti-mouse IgG (H + L) secondary antibody (Jackson ImmunoResearch Inc., West Grove, PA, USA) for 60 min at 24–28 °C. Cells were mounted with ProLong Gold Antifade Mountant with DAPI, and fluorescent images were acquired with a BZ-9000 microscope. The percentages of influenza nucleoprotein-positive cells were analysed by BD FACSCantoII flow cytometer and FlowJo software version 10.

### Statistical analysis

Data are presented as mean + standard error of the mean (SEM). Statistical significance was calculated using GraphPad Prism software version 5 (San Diego, CA, USA). Statistical tests and specific *p*-values are indicated in the figure legends.

## Supplementary information

Supplementary Information 1.

## Data Availability

The datasets generated during and/or analysed during the current study are available from the corresponding author on reasonable request.
