# Supplementary material for: Cell-penetrating peptide-mediated cell entry of H5N1 highly pathogenic avian influenza virus
Source: Sci Rep. 2020 Oct 22;10:18008. doi: 10.1038/s41598-020-74604-w (PMC7582914; doi:10.1038/s41598-020-74604-w)
Supplement: Supplementary file 1 — Supplementary Information 1. [file 41598_2020_74604_MOESM1_ESM.docx]

**Supplementary Information**

**Cell-penetrating peptide-mediated cell entry of H5N1 highly pathogenic avian influenza virus**

**Naoki Kajiwara^1, 4^, Namiko Nomura^1, 4^, Masako Ukaji^1, 4^, Naoki Yamamoto^2^, Michinori Kohara^2^, Fumihiko Yasui^2^, Yoshihiro Sakoda^3^, Hiroshi Kida^3^, and Futoshi Shibasaki^1, 5, *^**

**Supplementary methods**

**Cells**

HeLa, NIH/3T3, and J774A.1 cells were maintained in DMEM supplemented with 10% FBS, 100 unit/mL penicillin, 100 μg/mL streptomycin, 2 mM L-glutamine, and 1 mM sodium pyruvate. Jurkat, Raji, U937, THP-1, and HL-60 cells were maintained in RPMI-1640 supplemented with 10% FBS, 100 unit/mL penicillin, and 100 μg/mL streptomycin. These cells were cultured at 37 ºC in 5% CO_2_.

**Flow cytometric analysis**

HeLa (2.0 x 10^4^ cells), A549, NIH/3T3 (1.0 x 10^4^ cells), and MDCK (5 x 10^3^ cells) cells were cultured in 24-well plates for two days. After washing with D-PBS, these cells were incubated with 10 μg/mL FITC-labelled peptides or serum-free Opti-MEM for 60 min at 37 ºC and harvested using 0.25% trypsin-EDTA treatment. J774A.1, Jurkat, Raji, U937, THP-1, and HL-60 cells (2.5 x 10^5^ cells) were incubated with 10 μg/mL FITC-labelled peptides or Opti-MEM in 96-well plates for 60 min, then treated with 0.1% trypsin-EDTA for 10 min at 37 ºC to remove peptides bound on cell surface. For time course experiments, KU812 cells were incubated with 10 μg/mL FITC-labelled peptides at 37 ºC for 30, 60, 120, 180 and 240 min. For experiments with dimerised peptides, KU812 cells were incubated with 10 μg/mL non-treated or DMSO-treated peptides or Opti-MEM in 96-well plates for 60 min, then treated with 0.1% trypsin-EDTA for 10 min at 37 ºC. For experiments using sialidase, KU812 cells were incubated with 0.5 unit/mL sialidase from *Streptococcus 6646K* (SEIKAGAKU BIOBUSINESS Corp., Tokyo, Japan) for 60 min at 37 ºC. For experiments with trypsin pre-treatment, KU812 cells were incubated with 5, 10, and 50 μg/mL TPCK-trypsin (Sigma-Aldrich) for 30 min at 37 ºC. KU812 cells were subjected to FITC-conjugated peptides and geometric MFI of FITC in 7-amino-actinomycin D-negative viable cells was analysed using BD FACSCantoII flow cytometer and FlowJo software version 10.

**Disulphide bond formation**

Disulphide-bound homodimers were formed by incubating the peptide overnight at 24-28 ºC with 20% DMSO. Peptides were separated using SDS-PAGE (4-12% gradient gel) and FITC signals were detected using the LAS-4000 fluorescence imaging system (Fuji Film, Tokyo, Japan). For mass spectrometry, non-labelled peptides were used. Samples were purified using ZipTipC18 pipette tips (Merck Millipore, Billerica, MA, USA). The purified samples were mixed with matrix, alpha-Cyano-4-hydroxycinnamic acid, and then spotted onto a stainless-steel target plate. Analyses of peptide masses were performed using MALDI-TOF-MS autoflex maX (Bruker Daltonics, Germany).

**Sialic acid-galactose levels on cell surface**

KU812 cells were stained with FITC-conjugated MAA or SNA lectin at 24-28 ºC for 15 min. Data were acquired using BD FACSCantoII flow cytometer and analysed using FlowJo software version 10.

**Cell viability**

Lec8 cells (2 x 10^4^ cells) were cultured for three days in 12-well plates. After medium change, cells were further cultured for 24 and 48 h at 37 ºC in 5% CO_2_. For experiments with macropinocytosis inhibitor, Lec8 cells were treated with vehicle or EIPA (10, 20 and 40 μM) at 37 ºC for 8 h. Total and live cell numbers were counted using trypan blue dye (Gibco) and cell viability was calculated.

**Virus infection assay**

CHO-K1 and pgsA-745 cells (2 x 10^4^ cells) were cultured for three days in 12-well plates. These cells were incubated with reassortant viruses at MOI 10 for 60 min at 37 ºC. After washing, cells were cultured in α-MEM containing 10% FBS, 100 unit/mL penicillin and 100 μg/mL streptomycin for 8 h. The infected cells were fixed in 4% paraformaldehyde and were stained with mouse anti-influenza A nucleoprotein monoclonal antibody and Alexa Fluor 488-conjugated mouse IgG secondary antibody. Data were acquired by BZ-9000 microscope and BD FACSCantoII flow cytometer.

**Supplementary figures**

**
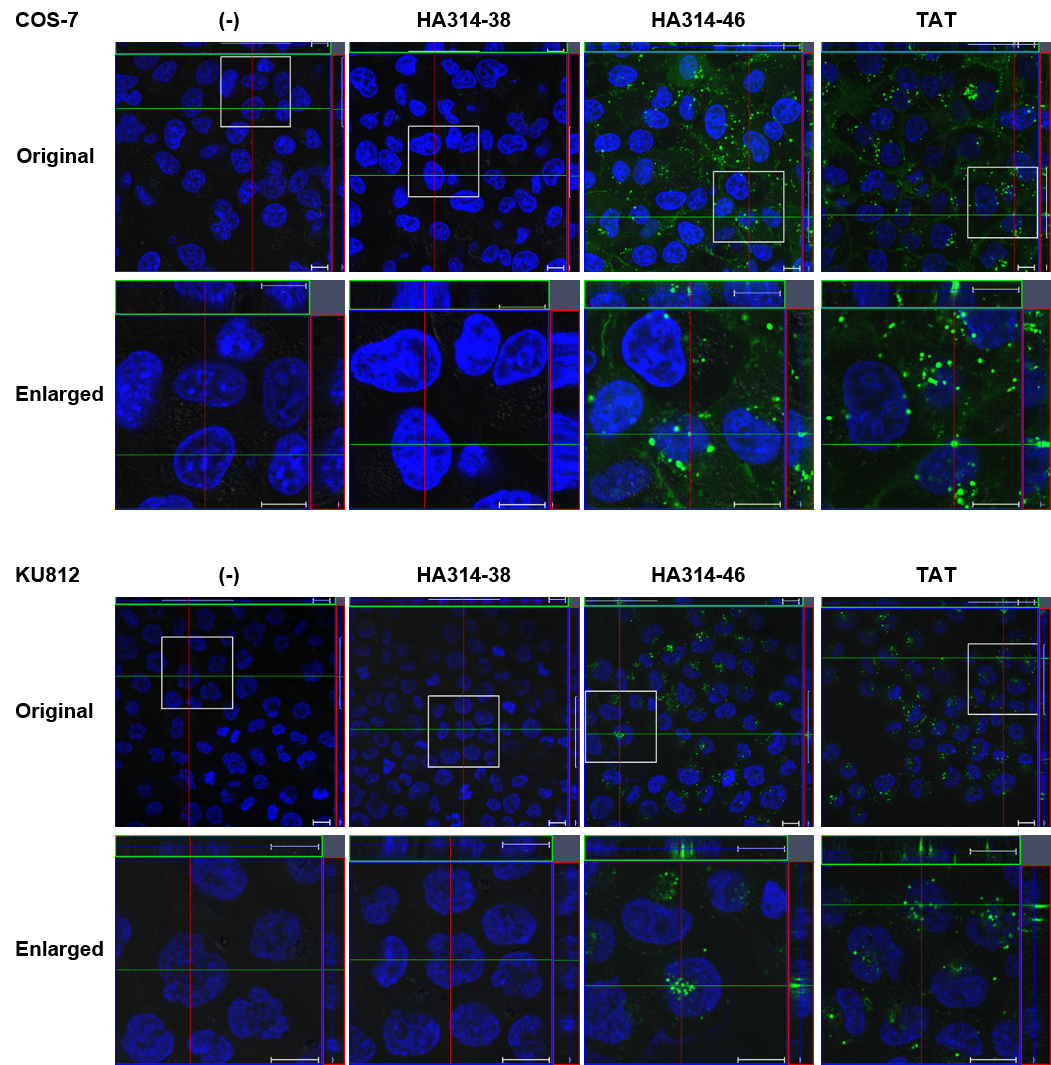
**

**Supplementary figure S1. HA314-46 and TAT peptides are localised in the cytoplasm and nucleus.**

COS-7 and KU812 cells were incubated with 10 μg/mL of FITC-conjugated peptides at 37 ºC for 60 min. To confirm the nuclear localisation of peptide, confocal microscopic images were sectioned in the Z-axis and orthogonally projected. Representative images from three experiments are shown. Green, peptide; Blue, nucleus. Scale bar, 10 μm.

**
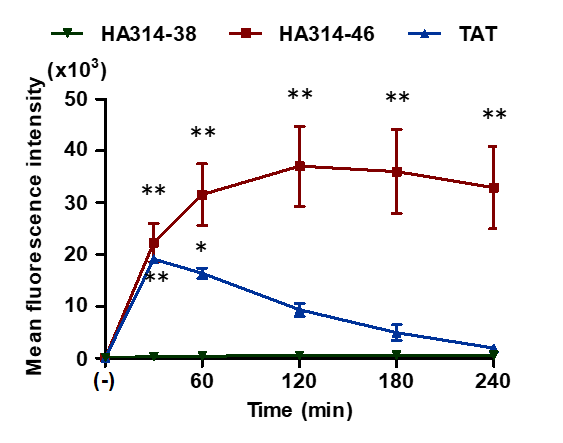
**

**Supplementary figure S2. HA314-46 peptide is rapidly internalised into KU812 cells.**

KU812 cells were incubated with 10 μg/mL of FITC-conjugated peptides at 37 ºC for 30, 60, 120, 180, 240 min. MFI of FITC in variable cells was assessed by flow cytometry. Data are shown as mean ± SEM (n = 3). Asterisks indicate significant increase by two-way ANOVA with Bonferroni's multiple comparison test. ***p* < 0.01; **p* < 0.05 vs (-).

**
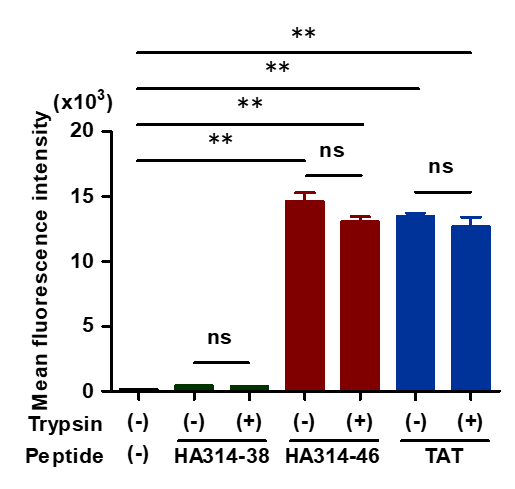
**

**Supplementary figure S3. Peptide internalisation is validated by the post-treatment with trypsin.**

KU812 cells were incubated with 10 μg/mL of FITC-conjugated peptides for 60 min at 37 ºC. Then, cells were treated in the absence or presence of 0.1 % trypsin-EDTA for 10 min at 37 ºC to remove cell surface-bound peptides. MFI of FITC in variable cells was determined by flow cytometry. Data are shown as mean + SEM (n = 3). Asterisks indicate significant increase by one-way ANOVA with Bonferroni's multiple comparison test. ***p* < 0.01; ns, not significant.


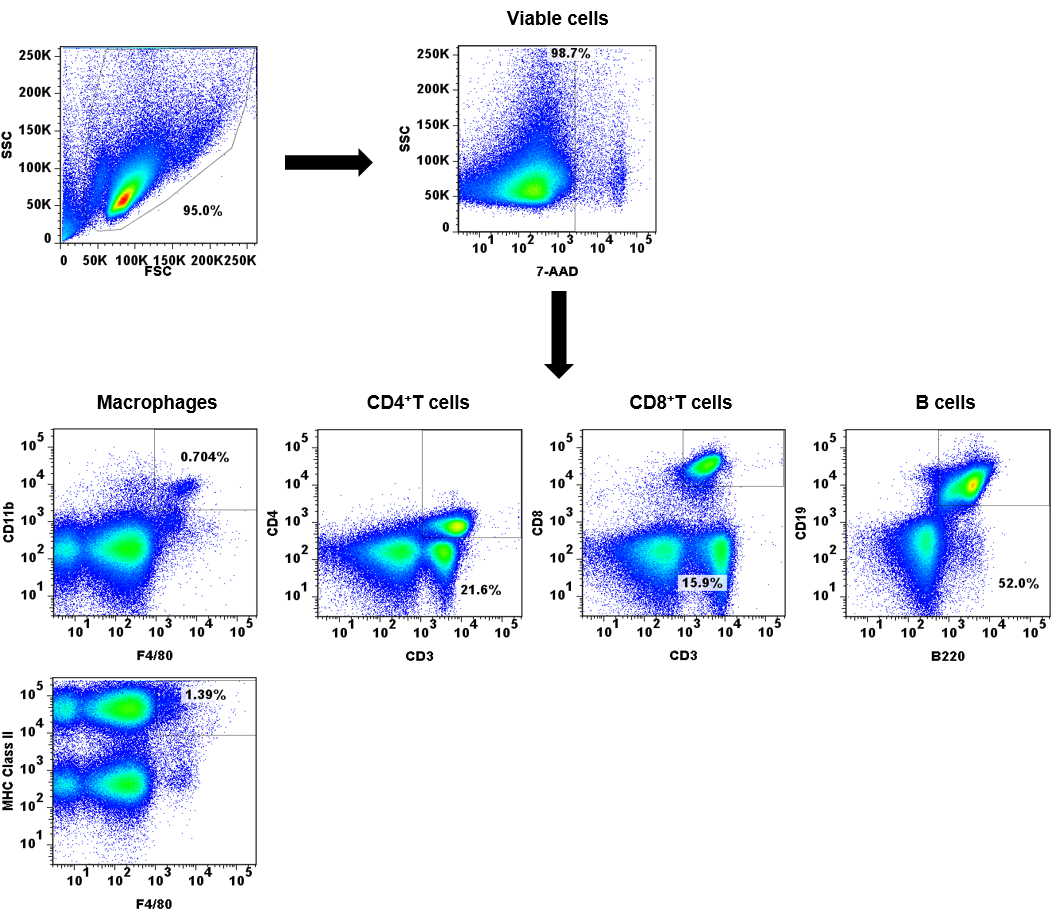


**Supplementary figure S4. Mouse splenocytes are separated by appropriate cell surface markers.**

After FITC-labelled peptides and 0.1% Trypsin-EDTA treatments, splenocytes were stained with antibodies against CD8α, CD19, B220, F4/80, I-A/I-E, CD3e, CD4 and CD11b. Macrophages, CD11b^+^F4/80^+^MHC class II^+^; CD4^+^T cells, CD3^+^CD4^+^; CD8^+^T cells, CD3^+^CD8^+^; B cells, CD19^+^B220^+^. Representative dot-plots from four experiments are shown.


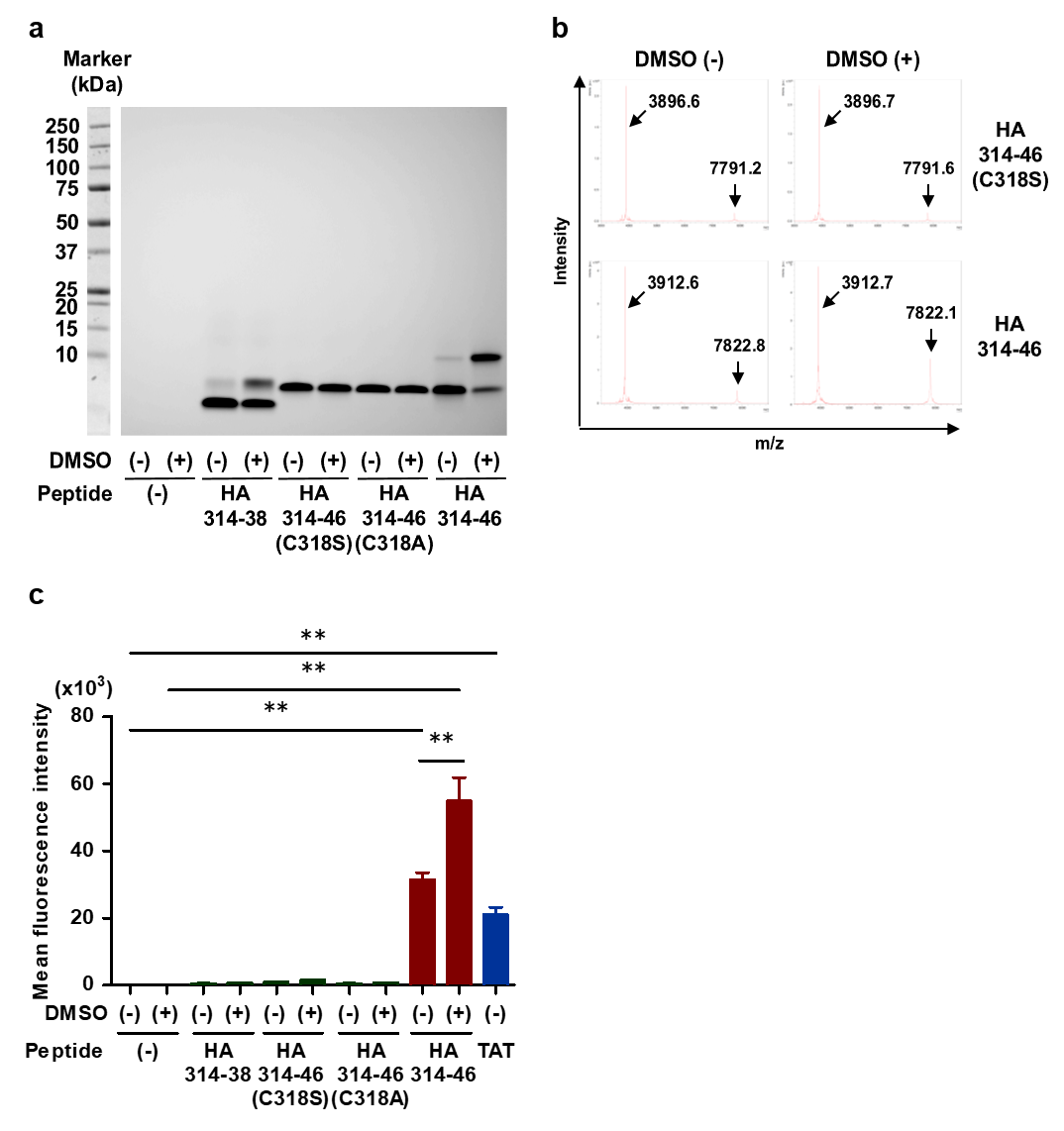


**Supplementary figure S5. HA314-46 internalisation is also observed in homodimers.**

(**a, b**) Disulphide bond formation by DMSO oxidation. HA peptides were dimerised by incubating overnight at 24-28 ºC in the presence of 20% DMSO. Dimerisation was confirmed using SDS-PAGE (**a**) and mass spectrometry (**b**). Molecular weight, HA314-46; 3911.6, HA314-46 (C318S); 3895.5 (**c**) Cell-penetrating activities of the DMSO-oxidised HA peptides. KU812 cells were incubated with non-treated or DMSO-treated HA peptides or TAT peptide (10 μg/mL) for 60 min at 37 ºC and then subjected to flow cytometric analysis to measure MFI of FITC. Data are shown as mean + SEM (n = 4). Asterisks indicate significant increase by one-way ANOVA with Bonferroni's multiple comparison test. ***p* < 0.01.


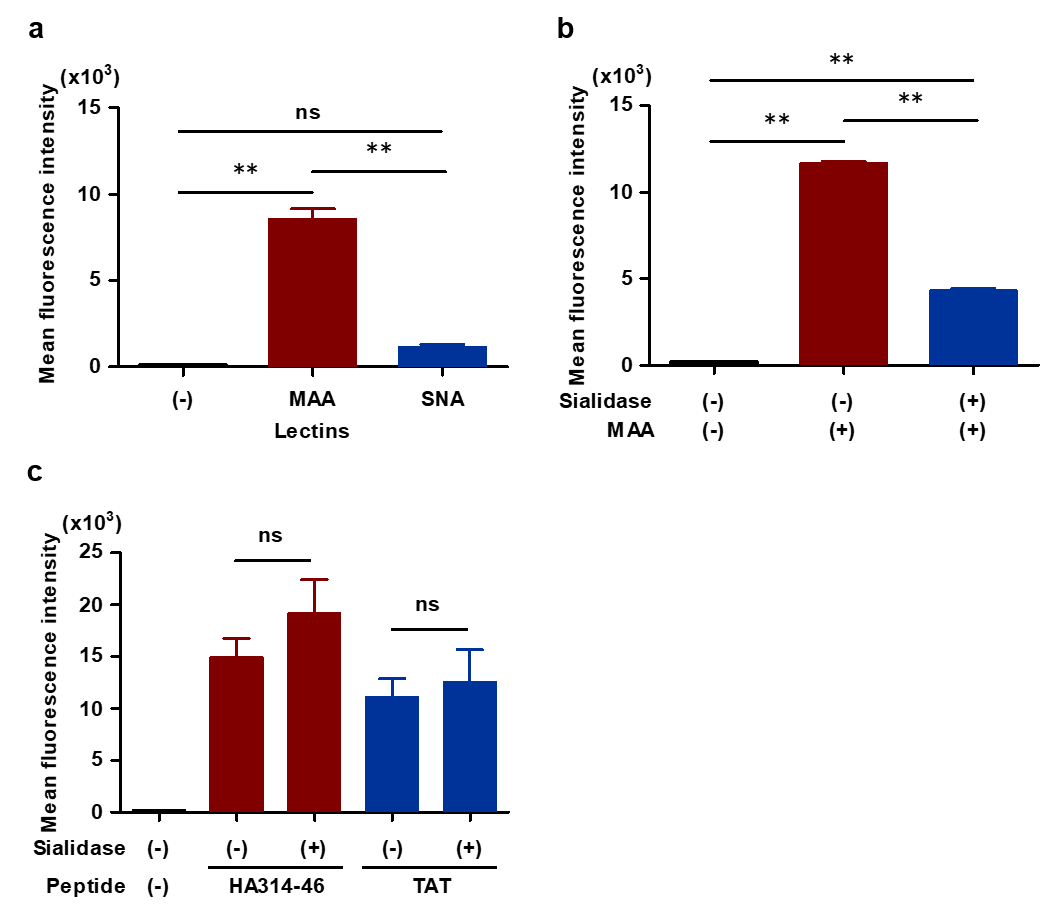


**Supplementary figure S6. HA314-46 activity is not affected by the pre-treatment of sialidase.**

(**a**) Cell surface levels of α(2,3)- and α(2,6)-linked sialic acid-galactose on KU812 cells. KU812 cells were stained with FITC-conjugated MAA or SNA lectin for 15 min at 24-28 ºC. (**b**) Effect of sialidase treatment on sialic acid-α(2,3)-galactose level. KU812 cells were treated with serum-free Opti-MEM or medium supplemented with 0.5 unit/mL sialidase from *Streptococcus 6646K* for 60 min at 37 ºC. Then, cells were stained with FITC-conjugated MAA lectin. (**c**) Effect of sialidase pre-treatment on HA314-46 peptide uptake. KU812 cells were treated with sialidase as described in (**b**). After washing, the cells were incubated with 10 μg/mL of FITC-conjugated HA314-46 or TAT peptide for 60 min at 37 ºC. Internalisation of the peptides was analysed using flow cytometry. Data are shown as mean + SEM (n = 3). Asterisks indicate significant difference by one-way ANOVA with Bonferroni's multiple comparison test. ***p* < 0.01; ns, not significant.


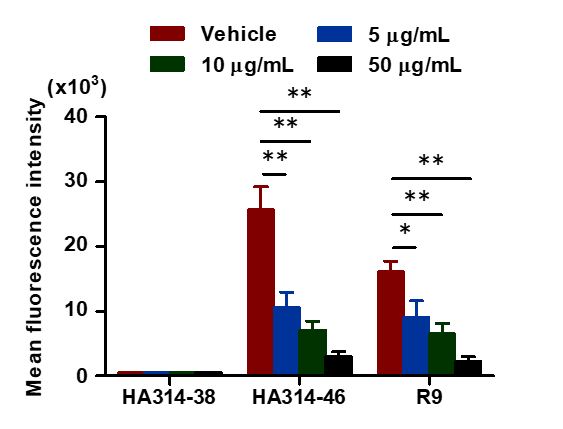


**Supplementary figure S7.** **TPCK-trypsin pre-treatment inhibits the cell-penetrating activity of the HA314-46 peptide.**

KU812 cells were pre-treated with TPCK-trypsin (5, 10, and 50 μg/mL) for 30 min at 37 ºC. After washing, the cells were incubated with 10 μg/mL of FITC-conjugated HA314-46 peptide for 60 min at 37 ºC and then MFI of FITC in variable cells was determined using flow cytometry. Data are shown as mean + SEM (n = 3). Asterisks show significant decrease by one-way ANOVA with Bonferroni's multiple comparison test. ***p* < 0.01; **p* < 0.05.


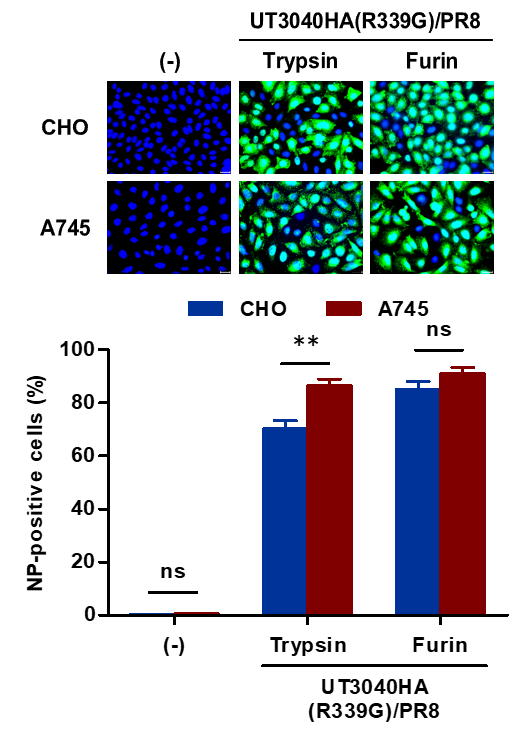


**Supplementary figure S8.** **The proportion of viral nucleoprotein-positive cells are comparable between pgsA-745 and CHO-K1 cells.**

CHO-K1 and pgsA-745 cells were incubated with trypsin- or furin-treated reassortant viruses at 37 ºC for 1 h at MOI of 10. After washing and incubation for 8 h, the cells were fixed and stained with anti-influenza A nucleoprotein monoclonal antibody. Localisation of the viruses and the ratio of nucleoprotein-positive cells were determined by fluorescent microscopy and flow cytometry, respectively. Representative images from four experiments are shown. Green; viral nucleoprotein, Blue; nucleus. Scale bar, 20 μm. Data are represented as mean + SEM (n = 4). Asterisk means significant difference by two-way ANOVA with Bonferroni's multiple comparison test. ***p* < 0.01; ns, not significant.


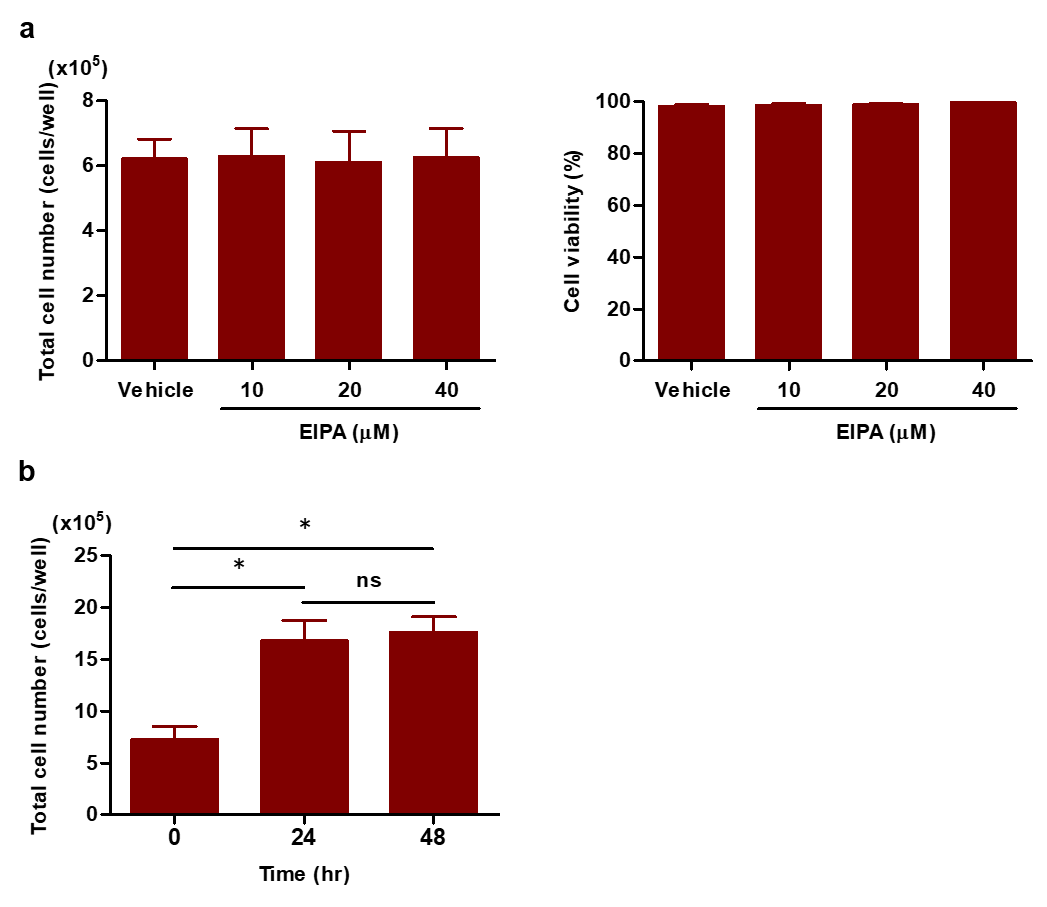


**Supplementary figure S9.** **The condition of cells is important for viral infection.**

(**a**) Effect of EIPA on cell number and viability. Lec8 cells were treated with vehicle or 10, 20 and 40 μM EIPA at 37 ºC for 8 h. (**b**) Total cell number of Lec8 cells for virus infection assay. Lec8 cells were further cultured for 24 and 48 h at 37 ºC in 5% CO_2_. Total cell number and cell viability were evaluated by trypan blue dye exclusion assay. Data are shown as mean + SEM (n = 3). Asterisks show significant increase by one-way ANOVA with Bonferroni's multiple comparison test. **p* < 0.05; ns, not significant.

**Supplementary Table S1. Cell-penetrating activities of HA314-38, HA314-46, or TAT peptides in various cultured cells**

|  | | Relative fluorescence intensity | | |
| --- | --- | --- | --- | --- |
| Cell lines | Cell types | HA314-38 | HA314-46 | TAT |
| A549  HeLa  KU812  Jurkat  Raji  U937  THP-1  HL-60  NIH/3T3  J774A.1  COS-7  MDCK | Epithelial-like cell  Epithelial-like cell  Myeloblast  T lymphocyte  B lymphocyte  Monocyte  Monocyte  Promyeloblast  Fibroblast  Macrophage  Fibroblast  Epithelial cell | 2.8 ± 0.2  2.7 ± 0.1  2.7 ± 0.1  2.4 ± 0.1  3.2 ± 0.3  1.9 ± 0.1  2.6 ± 0.1  1.6 ± 0.1  8.0 ± 0.4  5.1 ± 0.3  3.5 ± 0.1  4.0 ± 0.2 | 75.6 ± 4.0  95.4 ± 10.9  92.4 ± 0.8  35.4 ± 5.8  22.5 ± 6.2  22.0 ± 6.6  14.6 ± 0.6  6.2 ± 0.4  207.6 ± 12.3  105.4 ± 12.4  111.4 ± 4.0  94.1 ± 21.2 | 31.4 ± 1.8  90.0 ± 12.3  89.7 ± 3.4  32.7 ± 3.3  29.5 ± 4.1  43.5 ± 9.6  37.6 ± 7.5  25.6 ± 3.6  55.0 ± 23.1  68.0 ± 8.9  59.2 ± 7.3  58.7 ± 17.4 |

Cells were incubated with 10 μg/mL of FITC-conjugated peptides for 60 min at 37 ºC and then subjected to flow cytometric analysis. Values indicate the fold change of fluorescence intensity relative to that of untreated cells. Data is shown as means ± SEM (n = 3).
